# Seasonal alternation of the ontogenetic development of the moon jellyfish *Aurelia coerulea* in Maizuru Bay, Japan

**DOI:** 10.1371/journal.pone.0225513

**Published:** 2019-11-21

**Authors:** Kentaro S. Suzuki, Keita W. Suzuki, Emi Kumakura, Kana Sato, Yutaro Oe, Tasuku Sato, Hideki Sawada, Reiji Masuda, Yasuyuki Nogata

**Affiliations:** 1 Environmental Science Research Laboratory, Central Research Institute of Electric Power Industry, Abiko, Chiba, Japan; 2 Maizuru Fisheries Research Station, Field Science Education and Research Center, Kyoto University, Maizuru, Kyoto, Japan; 3 CERES Inc., Abiko, Chiba, Japan; 4 College of Bioresource Sciences, Nihon University, Fujisawa, Kanagawa, Japan; Evergreen State College, UNITED STATES

## Abstract

Outbreaks of moon jellyfish *Aurelia* spp. are frequently reported from many parts of the world’s coastal areas. *Aurelia* spp. canonically show a metagenetic life cycle in which planulae transform into sessile polyps, which can drastically increase in number through asexual reproduction. Therefore, their asexual reproduction has been recognized as one of the major causes of the outbreaks. *Aurelia* spp. also show direct development that lacks asexual reproduction during the polyp stage, which prevents us from understanding the mechanisms of its outbreaks. To clarify the seasonality of the metagenetic and direct-development life cycles of *Aurelia* sp. in Maizuru Bay, Japan, we conducted field observations and laboratory experiments throughout the year. Additionally, the two life cycle types were genetically analyzed to confirm that they belong to the single species *Aurelia coerulea*, which dominates in coastal waters in Japan. From July until October, *Aurelia coerulea* produced smaller eggs and planulae all of which developed into polyps. However, from December until May, larger eggs and planulae were produced and 90% of the planulae developed into planktonic ephyrae bypassing the sessile polyp stage. Our results demonstrated that a single species, *A*. *coerulea*, seasonally shifts between their two life cycle types at a water temperature threshold of 20°C in Maizuru Bay. The higher energy storage of larger planulae was suggested to enable the planulae to develop into ephyrae without external energy input through feeding during the polyp stage. The adaptive significances of the two life cycle types were also discussed.

## Introduction

Outbreaks of scyphozoan jellyfish have been frequently reported from many parts of the world’s coastal areas [1–3]. Among the scyphozoans, moon jellyfish *Aurelia* spp. are most common in temperate coastal waters, and outbreaks of *Aurelia* spp. can have severe effects on coastal ecosystems and human enterprises, such as fisheries and coastal power plant operations [1,4–6]. Therefore, mechanisms that cause the outbreaks of *Aurelia* spp. are of ecological and socio-economic interest.

**Fig 1:**
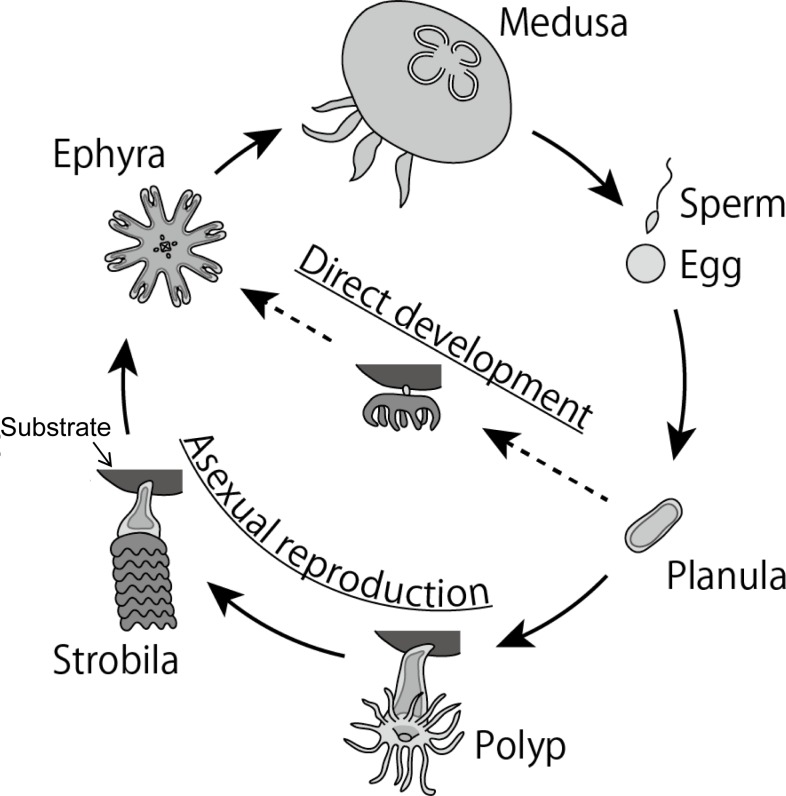
Two life cycle types of *Aurelia* spp. The life cycles are illustrated based on [10,12,19].

Population dynamics have been studied for many species of marine organisms based on their life cycles (e.g., [7–9]). Similar to other scyphozoans, *Aurelia* spp. canonically have both asexual/sessile and sexual/planktonic stages in the life cycle (Fig 1, reviewed in [10]). In the metagenetic life cycle, a fertilized egg develops into a ciliated non-feeding planula larva, which settles down on a suitable substrate and then forms an asexual polyp. Environmental stimuli, such as temperature decline [11,12], cause a polyp to metamorphose into a strobila with several transverse segments in the body column, giving rise to multiple planktonic ephyrae, which grow up into sexually reproductive adult medusae. *Aurelia* spp. can significantly increase in number through asexual reproduction (e.g. budding and strobilation [12,13]) during the sessile period. Therefore, asexual reproduction has been recognized as a key process for outbreaks (e.g., [1,14]), and thus environmental factors that control asexual reproduction, such as temperature, prey abundance, and light, have been well studied [15–18].

Besides the above-mentioned canonical life cycle (hereafter described as “metagenetic life cycle”), it is reported from Japan and Germany that *Aurelia* spp. have another type of life cycle which lacks an asexual polyp stage (Fig 1, [12,19–22]). Contrary to holoplanktonic scyphozoans, such as *Pelagia noctiluca* and *Periphylla periphylla*, *Aurelia* spp. planulae of this alternative life cycle temporarily settle on substrate, and one settled planula produces one ephyra [12,19]. Though there is such a difference of *Aurelia* spp. from the holoplanktonic scyphozoans, this alternative life cycle of *Aurelia* spp. has been described as “direct development” because of the lack of an asexual polyp stage [10,19,23,24]. In the present study, we utilize the term “direct development” to be consistent with previous studies. In the direct-development type life cycle, *Aurelia* spp. do not increase in number through asexual reproduction during the polyp stage, and thus the mechanisms of outbreaks should be different between the two life cycle types. However, little is known about the direct development of *Aurelia* spp. Planulae from the direct-development type, which were collected in Tsuruga Bay, Japan, were larger (500–700 μm in the major axis, [22]) than planulae that transformed into polyps in other regions (220–375 μm, [25,26]). In addition, the medusae of *Aurelia* sp. sexually reproduce mainly from winter to spring in Tsuruga Bay [19,27], which is contrary to the summer spawning of *Aurelia* spp. reported in many other regions (reviewed in [23]). Therefore, larger planulae and spawning in the cold season are hypothesized to be key characteristics of the direct development of *Aurelia* spp. However, the two life cycle types of *Aurelia* spp. have never been studied to test this hypothesis or elucidate the population dynamics.

To test the hypothesis of seasonal alternation between the two life cycle types in Maizuru Bay, Japan, field observations and laboratory experiments were conducted throughout the year. In Maizuru Bay, *Aurelia* sp. medusae occur throughout the year and produce planulae even in winter (R Masuda, personal communication). Additionally, *Aurelia* sp. medusae representing both life cycle types were genetically analyzed to confirm that they belonged to a single species, because the genus *Aurelia* contains many cryptic species [28–31]. The two life cycle types were discussed in terms of controlling factors in the environment and ecological significance in their adaptations.

## Materials and methods

### Ethics statement

Field observations in the present study were approved by the harbormaster of Maizuru Bay. *Aurelia* sp. is not an endangered or protected species, and no permits were needed to sample *Aurelia* sp. medusae in Maizuru Bay. Samplings of medusae and zooplankton did not involve endangered or protected species.

### Maizuru Bay

**Fig 2:**
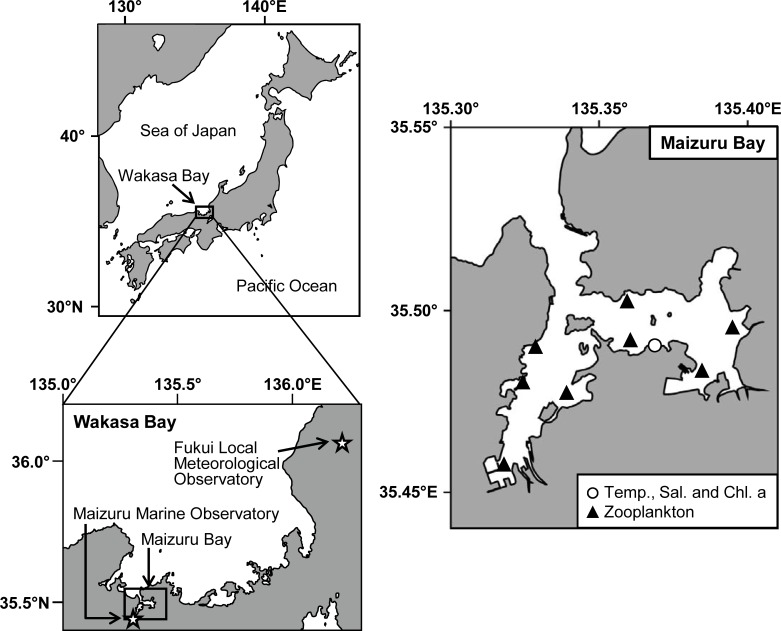
Study area. Stars on the left bottom map indicate observatories from which global horizontal irradiance data were obtained. A circle and black triangles on the right map indicate stations where environmental parameters were observed.

Maizuru Bay is a typical semi-enclosed water area with a surface area of approximately 23 km^2^, located in the western part of Wakasa Bay, Japan (Fig 2). The mean sea bottom depth is approximately 20 m, while the deepest area is 30 m deep. The tidal range is small (maximum: approximately 30 cm), because the Sea of Japan is connected to the Pacific Ocean through the narrow and shallow Korea, Tsugaru, and Soya Straits. Several rivers discharge into the bay. In this area, precipitation is rather high from summer to winter due to the rainy season, typhoon passages, and heavy snow on the Sea of Japan side.

### *Aurelia* sp. collections

From November 2016 to January 2018, *Aurelia* sp. medusae were collected monthly from aggregations using a ship, the *RV Ryokuyo Maru* of Kyoto University, or from the pier of Maizuru Fisheries Research Station, Kyoto University. Aggregations of *Aurelia* sp. were found by visual surveys of the surface water or on the display of a fishfinder HDS-12 Gen2 Touch with a transducer of 200 kHz (Lowrance Electronics Inc., Tulsa, OK, USA). In each month, at least 21 medusae were collected with hand nets for the surface aggregations or with an obliquely towed conical net for the sub-surface aggregations (mouth diameter of 0.8 m, length of 2.3 m and mesh size of 5 mm). The bell diameters of collected medusae were measured and it was visually observed whether they bore planulae in brood sacs on the oral arms. In principle, samples of the planula, ovary, and tentacle at the bell margin were taken from five planula-bearing females in each month. If no medusa bore planulae, five females with bell diameters of > 14 cm, which is large enough for spawning in Maizuru Bay, were sampled. The planulae were rinsed thoroughly from the selected females with seawater. Collected planulae were rinsed with filtered seawater (0.22 μm) on a 60 μm mesh to remove mucus, and were then stored in plastic bags with the filtered seawater for transportation to the laboratory. The plastic bags were stored in thermos bottles, which were installed in a portable cooler to maintain a constant temperature during transportation. The mean water temperature change was 1.2 ± 0.8°C (mean value ± SD, the same applies hereinafter) during the transportation of approximately 16 h. In the laboratory, planulae were stored in an incubator at the same temperature as the *in situ* water temperature for several hours until further observations and developmental experiments. The collected ovaries were preserved in 5% buffered formalin solution for further observations of the oocytes. Tentacles were preserved in 99% ethanol for further DNA analysis. The bell diameter data were classified into two groups based on results of the developmental experiments: samples from December 2016 until May 2017 when the direct development occurred (direct-development period) and from June 2017 until October 2017 when all planulae developed into polyps (metagenetic period). Then, the bell diameters were compared between the periods of the two life cycle types with Welch’s *t*-test due to unequal sample numbers and unequal variances. In the present study, all statistical tests were carried out using R 3.4.3 [32].

### Planula and oocyte observations

To estimate the total number of planulae per female medusa, which has been utilized as an indicator of egg production [33–35], the numbers of planulae in three replicates of 1-ml subsamples were counted for each sample. Twenty planulae for each female were measured for the length of the major and minor axes under a dissecting microscope. The volume of the planula was calculated from the length of the major and minor axes with an assumption in which the planula was in a spheroid shape. One of four ovaries was selected for each female. The selected ovaries were weighed, and then the weight was multiplied by four to estimate the total ovary weight of each female, as the weight of each ovary accounted for approximately 25%, ranging from 20 to 30%, of the total ovary weight (n = 5). The diameter of the oocytes (up to 200 in number), which are in a sphere-like shape, were measured under a dissecting microscope. The numbers of planulae per medusa and per ovary weight, the lengths of the planula, and the diameters of the top 5% of oocytes were compared between the direct-development and metagenetic periods using Welch’s *t*-test due to unequal sample numbers and unequal variances. The volume of the planulae and the top 5% of oocytes, i.e., most matured oocytes, were also compared in each month.

### Environmental parameters

To relate the seasonality of the two life cycle types to environmental parameters, global horizontal irradiance, water temperature, salinity, chlorophyll fluorescence, and copepod abundance data were obtained. As weekly averaged global horizontal irradiance data have been unavailable at Maizuru Marine Observatory since March 2013, they were obtained from Fukui Local Meteorological Observatory, which is approximately 100 km away from Maizuru Bay (Fig 2). Weekly averaged global horizontal irradiance at the two observatories from January 2000 to March 2013 were highly correlated (r = 0.95), and thus the data from the Fukui Local Meteorological Observatory can be applicable to Maizuru Bay. The water temperature, salinity, and chlorophyll fluorescence were measured semi-weekly with the AAQ-RINKO (JFE Advantech Co., Ltd., Nishinomiya, Japan) at 0.1 m depth intervals from the observational pier of Maizuru Fisheries Research Station, Kyoto University (bottom depth: approximately 7 m, Fig 2). For water temperature and salinity, the mean surface and bottom layer values were calculated from data of 0.1–1.0 m below the surface and those of 1.0–2.0 m above the bottom, respectively. Chlorophyll fluorescence data were averaged throughout the water column.

Zooplankton were collected with vertical tows of a cylindrical-conical net (mouth diameter of 0.3 m, length of 1 m and mesh size of 100 μm) from near the bottom to the surface at eight stations monthly from November 2016 to January 2018, except for August and September 2017, when the ship was unavailable (Fig 2). The volume of water filtered through the net was calculated from the length of the wire and the area of the net mouth. Zooplankton samples were preserved in 5% buffered formalin solution for later identification under a dissecting microscope. In the laboratory, zooplankton samples were divided into subsamples using a wide-bore pipette immediately after thorough mixing by pipetting. The subsamples were observed until the number of zooplankton exceeded 200 individuals. Then, zooplankton were identified to the family or genus level and the sizes were measured individually by length. In the present study, copepod biomass was used as a proxy of the availability of prey zooplankton for *Aurelia* sp., because copepods are a major prey of *Aurelia* spp. (e.g., [36,37]). The carbon amount of each copepod taxon was calculated from the size according to Uye [38], and then data from the eight stations were averaged monthly.

### Observation of ontogenetic development

To observe the development from planulae to the further stages, we incubated the collected planulae under darkness at the same temperature as the *in situ* water temperature. In the incubation, for each collected female, we first put approximately 100 planulae into a 24-well plate filled with 2 ml of filtered seawater (0.22 μm). Then, 20 settled planulae were randomly selected, and the others were removed to equalize the experimental settings among the wells. Ontogenetic development was observed daily under a dissecting microscope, and was categorized into three stages: settled individuals without tentacles, polyps, and liberated ephyrae. The weighted mean duration required for direct development from planula to ephyra (D_pla–ephy_) was calculated for each experiment with the following equation:

Dpla–ephy=∑nidintotal,

where *n*_*i*_ is the number of newly liberated ephyrae at day *i*, *d*_*i*_ is the days after the start of the experiment, and *n*_*total*_ is the total number of liberated ephyrae in the experiment. D_pla-ephy_ was examined in relation to water temperature. The total bell diameter (TBD) and central disc diameter (CDD) of the newly liberated ephyrae were measured under a dissecting microscope, according to Straehler-Pohl & Jarms [39].

### DNA sequencing

The mitochondrial cytochrome *c* oxidase subunit I (COI) gene has been utilized to identify sibling species of *Aurelia* (e.g., [31,40]). To compare haplotypes of *Aurelia* sp. in Maizuru Bay between the two life cycle types and also to haplotypes of *Aurelia coerulea*, which has been treated as *Aurelia* sp. 1 and is distributed along the coast of Japan [31], a partial region of the COI gene was chosen for analysis. Total DNA was extracted from the ethanol-preserved tentacles of female medusa using the DNeasy Blood & Tissue Kit (Qiagen Inc., Hilden, Germany) according to the manufacturer’s manual. A partial region of the COI gene was amplified using the forward primer of HCO2198 (5′-taaacttcagggtgaccaaaaaatca-3′, [41]) and the reverse primer of AaCOIi-L (5′-gcccgtyytaataggrgggtttgg-3′, [40]) in PCR according to Matsumura et al. [42]. Direct sequencing was performed on the ABI 3500 Genetic Analyzer (Thermo Fisher Scientific, Waltham, MA, USA) using the HCO2198 primer and BigDye Terminator v3.1 Cycle Sequencing Kit (Thermo Fisher Scientific Inc., Waltham, MA, USA). The nucleotide sequence data were checked visually in Sequencher v5.4.6 (Gene Codes Corporation, Ann Arbor, MI, USA), and then misreads were corrected. The COI sequences from Maizuru Bay were classified into two groups based on the life cycle type (direct-development or metagenetic period). We obtained COI sequences of *A*. *coerulea*, which were collected in the North Pacific, from GenBank according to Scorrano et al. [31]: **AY903080**, **AY903168**, **EU010386**. Multiple COI sequences were aligned using Clustal W [43]. A COI phylogenetic tree was constructed by the maximum likelihood method and Kimura 2-parameter model using MEGA X [44,45]. Statistical support for tree nodes was determined by bootstrap analysis of 1000 replicates. A COI sequence of *Aurelia limbata* (**AY903189**), which is the most closely related species to *A*. *coerulea* [40], was used as an outgroup.

## Results

### Environmental parameters

**Fig 3:**
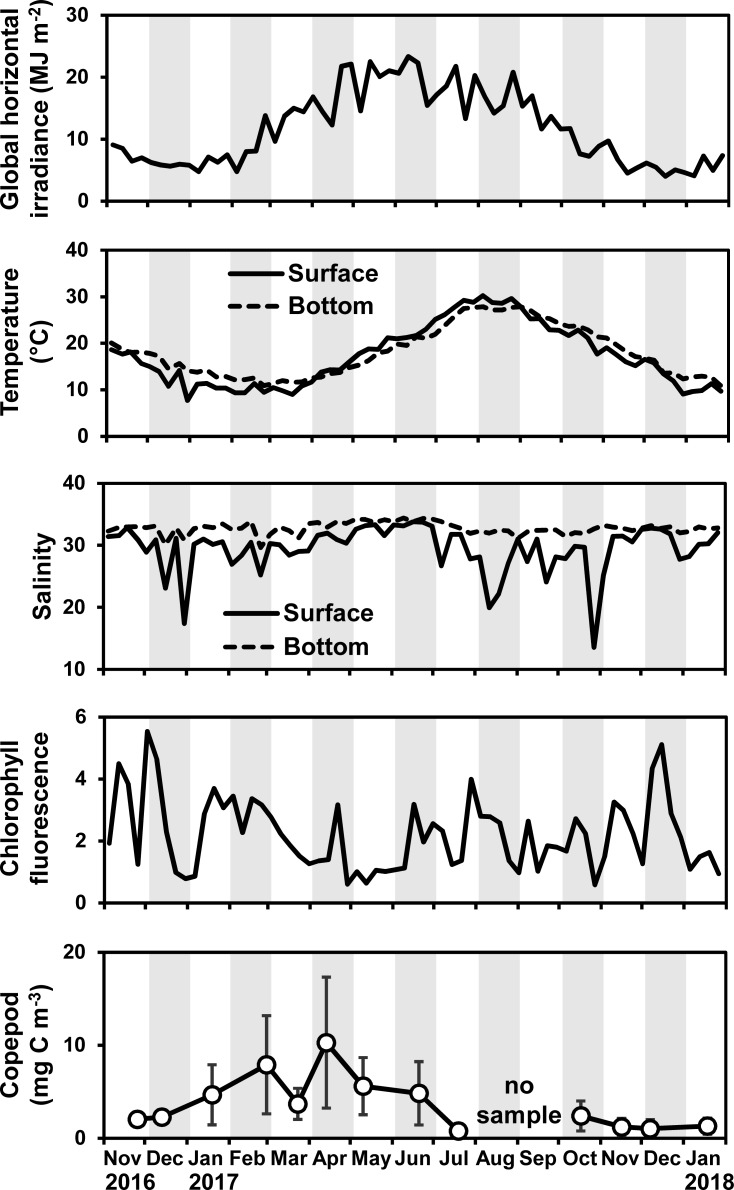
Temporal variations in environmental parameters. Chlorophyll fluorescence was averaged throughout the water column. Error bars in the copepod density indicate SD.

The global horizontal irradiance was low (< 10 MJ m^-2^) from December until March and peaked in June (23 MJ m^-2^, Fig 3). The water temperature at the bottom layer showed the minimum (approximately 11°C) and maximum values (approximately 28°C) in February and August, respectively (Fig 3). The water temperature at the surface layer showed a sharp decline in winter due to snow. The salinity at the bottom layer was almost constant (32 to 33) throughout the observation, while the surface salinity often decreased in the rainy and snowy seasons. The surface salinity dropped in response to heavy precipitation incidental to typhoons (August 7, September 17, and October 22 in 2017; according to Japan Meteorological Agency). Chlorophyll fluorescence exhibited peaks repeatedly through the year without a clear seasonal trend (Fig 3). Copepod biomass was higher from January until June than in other months (Fig 3).

### Development from planulae

**Fig 4:**
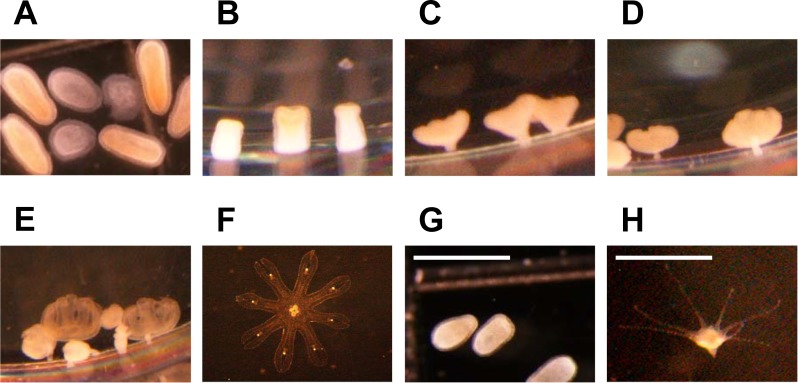
Two types of ontogenetic development of planulae in Maizuru Bay. Transformation process of the direct-development type from planula (A) to ephyra (F). The images B–F were taken 2, 6, 10, 12, and 15 days after the onset of incubation at 10°C, respectively. Transformation process of the metagenetic type from planula (G) to polyp (H). The image H was taken 7 days after the onset of incubation at 28°C. In B–E and H, *A*. *coerulea* attached to the wall of 24-well plates located in the lower part of the pictures. The scale bars indicate 1 mm.

**Fig 5:**
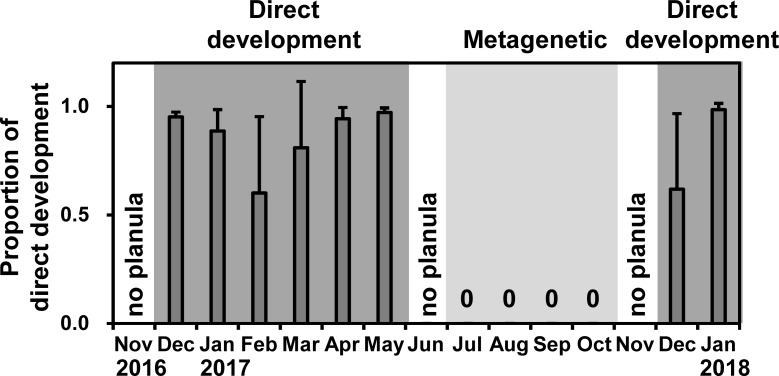
Temporal variations in proportion of direct development from planulae. Bars and error bars in the top figure are mean values and SD. No planula indicates that no female bore planulae. Dark and light shades indicate the direct-development and metagenetic periods, respectively.

**Fig 6:**
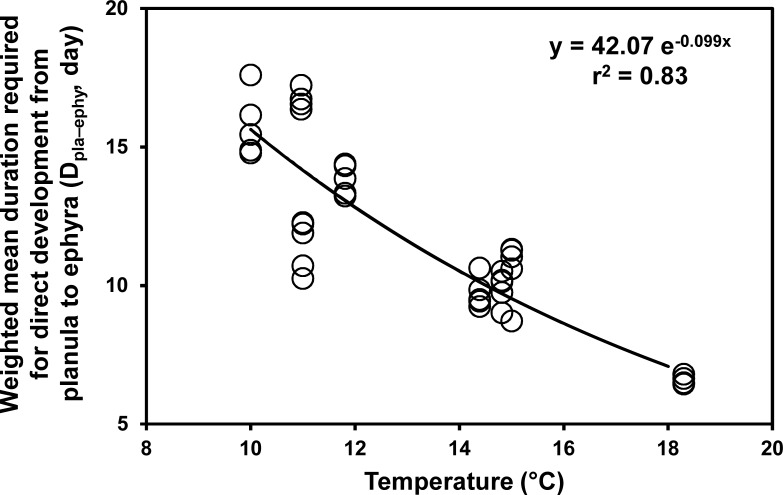
Duration required for direct development from planula to ephyra in relation to temperature.

A high proportion of planulae (approximately 90%) developed directly into ephyrae from December until May (direct-development period), while all planulae developed into polyps from July until October (metagenetic period, Figs 4 and 5). In June and November, the ontogenetic development of planulae was not observed, because no medusae bore planulae. D_pla–ephy_ declined exponentially with temperature increase, and the mean values at 10.0°C and 18.3°C were 16.7 days and 6.6 days, respectively (Fig 6). The mean total bell diameter (TBD) and central disc diameter (CDD) of the newly liberated ephyrae were 2.5 ± 0.4 mm and 0.9 ± 0.1 mm, respectively.

### Seasonal variations in medusae, oocytes, and planulae

**Fig 7:**
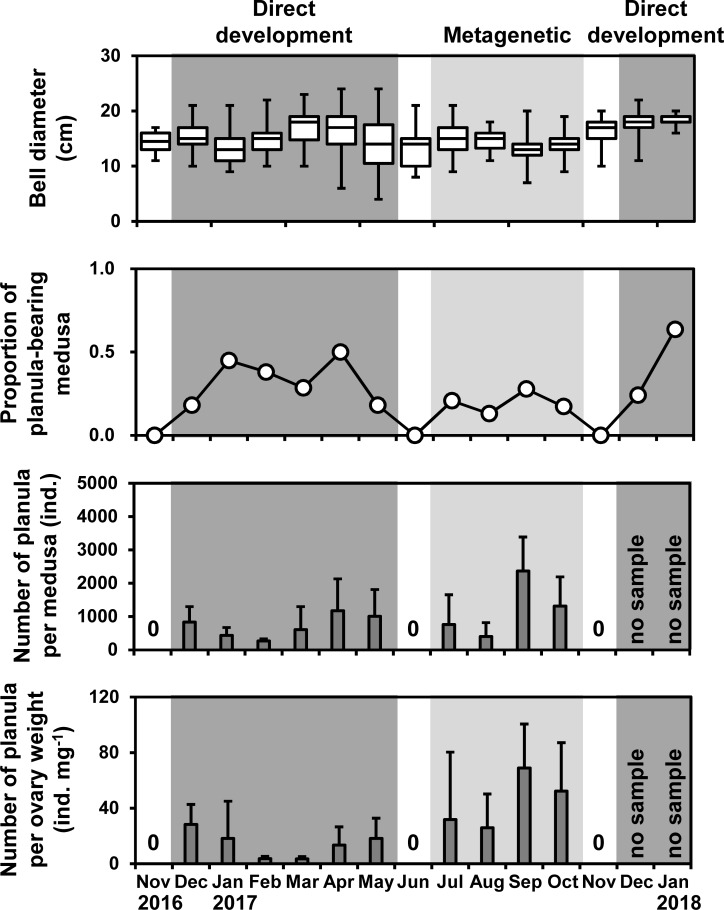
Temporal variations in the bell diameter of medusae, proportion of planula-bearing medusa, and the number of planulae per medusa and per ovary weight. In the top figure, boxes and bars in boxes indicate the first and third quartile and median values, respectively, and bars outside boxes indicate the minimum and maximum values. Bars and error bars in the bottom figure are mean values and SD, respectively. Dark and light shades indicate the direct-development and metagenetic periods, respectively.

The bell diameters of medusae were almost constant throughout the study period, being approximately 15 cm on average (Fig 7). Proportions of small medusae with bell diameters of < 10 cm were higher in May and June (> 10%) than in the other months (< 5%). The bell diameters of medusae were not significantly different between the direct-development and metagenetic periods (Fig 7; *P* = 0.25, Welch’s *t*-test). The proportion of planula-bearing medusa was 13% to 64% in the direct-development and metagenetic periods, while no medusae bore planulae in June and November (Fig 7). The number of planulae per medusa was not significantly different between the direct-development and metagenetic periods (Fig 7; direct-development period: 736 ± 731 planulae medusae^-1^; metagenetic period: 1028 ± 810 planulae medusae^-1^; *P* = 0.50, Welch’s *t*-test). In contrast, the number of planulae per ovary weight was significantly higher in the metagenetic period than that in the direct-development period (Fig 6; direct-development period: 10.6 ± 10.0 planulae mg^-1^; metagenetic period: 44.2 ± 24.5 planulae mg^-1^; *P* < 0.01, Welch’s *t*-test).

**Fig 8:**
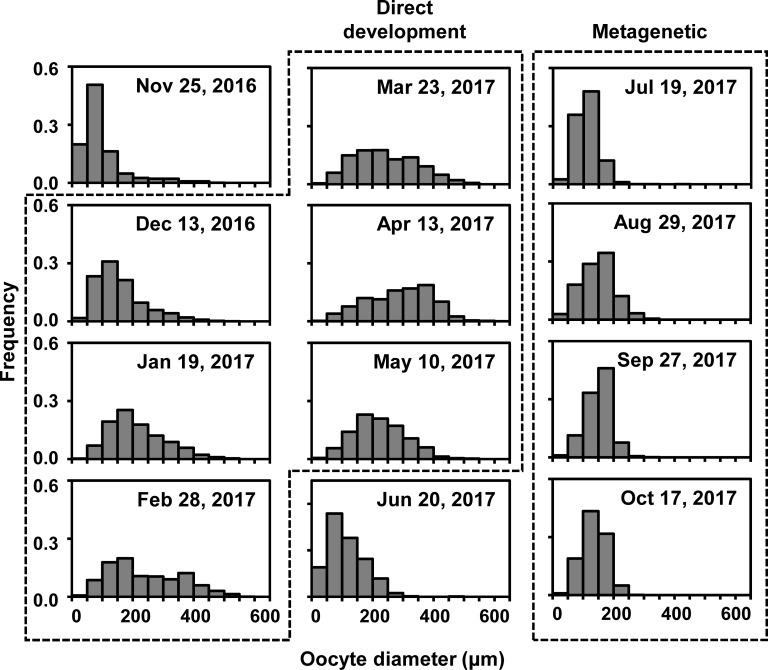
Seasonal change in oocyte diameter. Two boxes with dashed lines indicate the direct-development and metagenetic periods.

**Fig 9:**
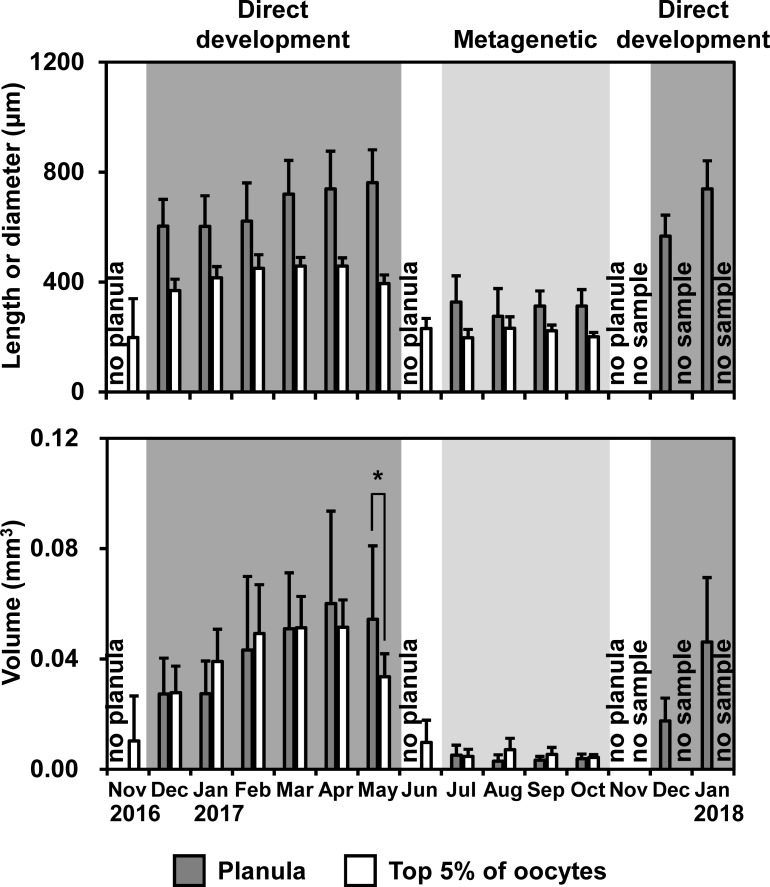
Temporal variations in the length of the major axis of the planulae, diameter of the top 5% of oocytes, and volume of the planulae and top 5% of oocytes. Bars and error bars are mean values and SD. * in the bottom figure indicates a significant difference (*P* < 0.05). No planula indicates that no females bore planulae. Dark and light shades indicate the direct-development and metagenetic periods, respectively.

During the metagenetic period, the monthly maximum of oocyte diameter ranged from 250 to 408 μm, while larger oocytes with a diameter of 504–571 μm were observed every month during the direct-development period (Fig 8). Although no planulae were found in November and June, oocytes in November and June were similar in size to those in December and July, respectively. The diameters of the top 5% of oocytes were significantly longer in the direct-development period (424 ± 37 μm) than in the metagenetic period (213 ± 17 μm, *P* < 0.01, Welch’s *t*-test; Fig 9). Similarly, the length of the major axis of the planula was significantly longer in the direct-development period (679 ± 142 μm) than in the metagenetic period (305 ± 75 μm, *P* < 0.01, Welch’s *t*-test). In both the planulae and the top 5% of oocytes, the volume was approximately 10-fold larger in the direct-development period than in the metagenetic period. Volumes of planulae and the top 5% of oocytes were not significantly different in each month (*P* > 0.05, Welch’s *t*-test), except for in May 2017 when the planulae were significantly larger than the top 5% of oocytes (Fig 9; *P* < 0.05, Welch’s *t*-test).

### COI sequences

**Fig 10:**
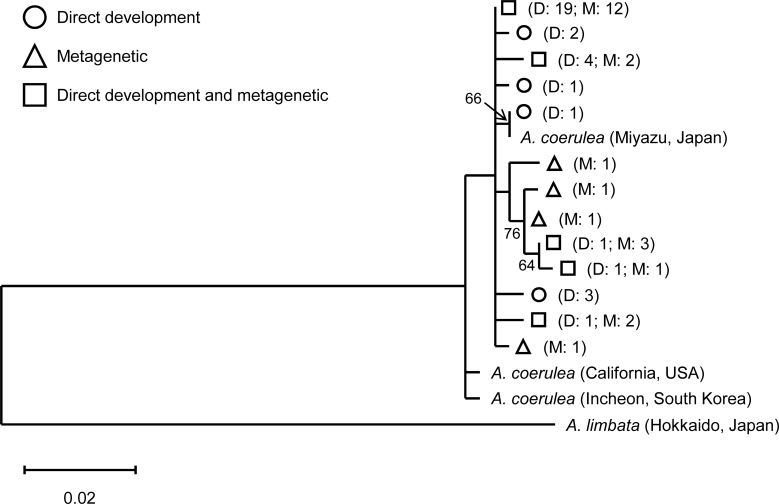
COI phylogenetic relationships among *Aurelia* of the two life cycle types in Maizuru Bay and *A*. *coerulea* from the North Pacific. The tree with the highest log likelihood (-862.22) is shown. Circle, triangle, and square indicate haplotypes that include specimens from the direct-development period, metagenetic period, and both periods, respectively. The letters following the symbols indicate the number of specimens from the direct-development (D) and metagenetic types (M). Bootstrap values > 50% are shown adjacent to branches. The scale bar indicates the number of substitutions per site. *A*. *limbata* was used as an outgroup.

From the COI sequence data in 374 bp, 13 haplotypes were detected from 57 individuals (Fig 10), most of which matched those of *Aurelia coerulea* previously reported from other Japanese regions [42,46,47]. In the COI phylogenetic tree, specimens from the two life cycle types did not show clear differences. The sequences of *Aurelia* from Maizuru Bay were much more similar to those of *A*. *coerulea* than to those of *A*. *limbata*.

## Discussion

### Seasonality of the two life cycle types of *A*. *coerulea* in Maizuru Bay

In Maizuru Bay, *Aurelia* sp. exhibited seasonal alternation of ontogenetic development, which corresponded closely to seasonal changes in the size of oocytes and planulae (Figs 5, 8 and 9). In addition, from the COI haplotypes, both types of *Aurelia* sp. in Maizuru Bay were suggested to belong to *A*. *coerulea* (Fig 10, hereafter described as *A*. *coerulea*). Previous studies have demonstrated that larger egg sizes are generally associated with direct development in marine invertebrates (reviewed in [48]). However, intraspecific variations in egg size and developmental type have rarely been reported, especially from a single population [49,50].

**Fig 11:**
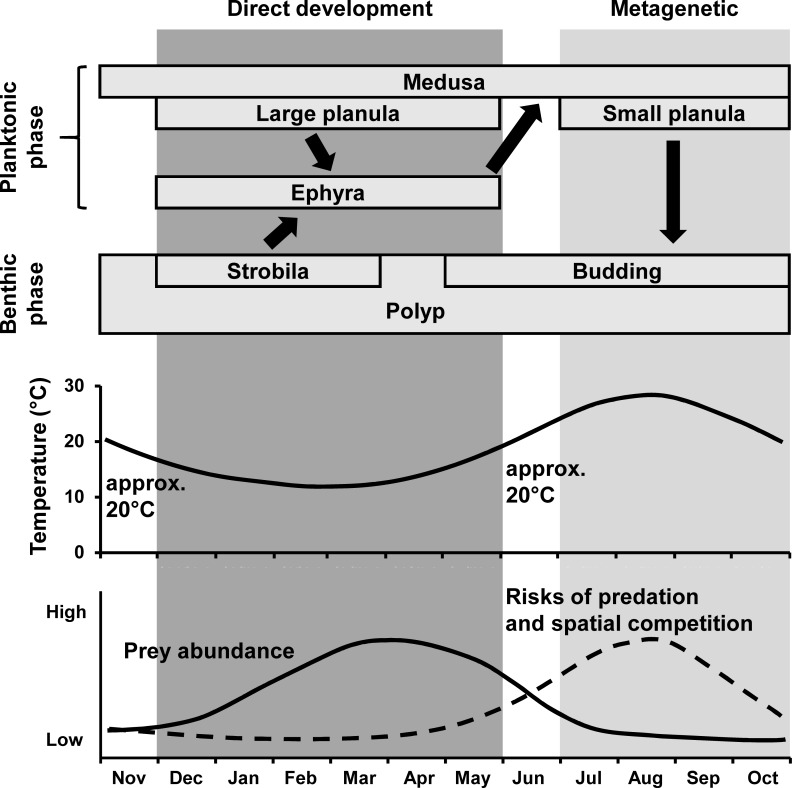
Schematic diagram of the seasonal life cycle of *A*. *coerulea* in Maizuru Bay. The timing of strobilation is inferred from Miyake et al., Makabe et al. and Watanabe & Ishii [53–55]. The copepod biomass is treated as prey abundance, with the assumption that it changes linearly from July to October. Risks of predation and spatial competition are inferred from the abundance and filtration rates of filter-feeding sessile organisms, according to Kim & Moon and Ishii & Katsukoshi [56,57].

The oocytes of the direct-development life cycle were reproduced by overwintering medusae (Figs 5 and 7). In Lake Nakaumi, a brackish lake in Japan, *A*. *coerulea* medusae are suggested to be able to overwinter when the water temperature in winter is higher than 5°C [51,52]. In the present study, the lowest water temperature in winter was higher than 5°C (Fig 3), and thus the water temperature was sufficient for the medusae to overwinter. In general, the longevity of *Aurelia* spp. medusa is from four months to less than two years [23]. Therefore, ephyrae, which are liberated through direct development from December until May, are suggested to develop into medusae and then reproduce in the following summer (metagenetic life cycle) and the next winter to spring (direct-development life cycle; Fig 11).

The canonical reproduction of the metagenetic life cycle was observed mainly in summer, similarly to the other regions (Fig 5, [23]). In Japan, ephyrae are liberated through strobilation mainly from December until March and then develop into young medusae in spring to early summer [53–55]. Assuming that the small medusae observed in May and June were new recruits, Maizuru Bay is similar in the timing of strobilation to the other regions in Japan. Therefore, the ephyrae liberated through strobilation are suggested to reproduce in the following summer (metagenetic life cycle) and the next winter to spring (direct-development life cycle) in the same way as in the direct-development life cycle (Fig 11).

### Environmental factors in relation to the two life cycle types

In the present study, *A*. *coerulea* clearly switched the two life cycle types. The planula size changed discontinuously in June and November when no medusae bore planulae (Figs 7 and 9). Although oocytes were not histologically analyzed in the present study, the top 5% of oocytes were considered to be fully matured (i.e., eggs), because there was no significant difference in volume between the top 5% of oocytes and planulae in most months of the year (Fig 9). Intraspecific variations in egg size are well reported from a wide variety of taxa, and mother size and environmental stresses are suggested to be the major causes (reviewed in [58]). The mother sizes of *A*. *coerulea* in the present study were almost constant throughout the year. Thus, in Maizuru Bay, *A*. *coerulea* is suggested to switch life cycle type due not to the mother size, but to the environmental changes in June and November (hereafter described as switching periods).

The environmental stimuli that cause the switch in egg size should be different between the direct-development and metagenetic periods and should also show changes in the switching periods. Salinity, prey availability and water temperature are reported as the major environmental stresses affecting the egg size of marine animals [58]. In the present study, neither the salinity nor copepod density showed significant changes in the switching periods (Fig 3), and thus salinity and prey availability are unlikely to be the causes. Negative correlation between water temperature and egg size is a well-known phenomenon for a number of fish and invertebrate species (e.g., [48,59,60]). In the bottom layer, where *A*. *coerulea* usually aggregates in Japanese coastal waters [61–63], water temperatures of 19.8–22.0°C and 17.1–21.1°C were observed in June and November, respectively (Fig 3). As the water temperature was different between the direct-development and metagenetic periods and showed similar values (approximately 20°C) in the switching periods, a water temperature of 20°C is suggested to be the threshold for switching the egg size and ontogenetic development of *A*. *coerulea* in Maizuru Bay.

### Egg size in relation to the two life cycle types

During the direct-development period, *A*. *coerulea* exhibited larger eggs than *A*. *coerulea* in metagenetic period (Fig 9). The carbon amount of the planula of *Aurelia* sp. with a major axis length of 200–320 μm was reported by [64] to be 0.36 μg, which is similar to the size of the metagenetic planulae in the present study (Fig 9). The carbon amount of the metagenetic planula is much smaller than that of the newly liberated ephyra in the present study (1.0 μg), estimated from the CDD with the allometric equation of Kamiyama [65]. This indicates that the metagenetic planulae do not store enough energy to transform directly to ephyrae. Assuming that the carbon amount of a planula is correlated to the volume, the planula during the direct-development period is estimated to contain approximately 4 μg C, which is even higher than the carbon amount of a newly liberated ephyra. Therefore, the high energy storage of the large eggs and planulae during the direct-development period is suggested to enable the planulae to develop into ephyrae without external energy input through feeding. From many studies on marine invertebrates, larger eggs are suggested to be necessary for direct development because of higher requirements for energy in early development (reviewed in [48]). Berrill [66] hypothesized that among scyphozoan jellyfish, larger eggs would be associated with direct development. Egg sizes of the holoplanktonic jellyfish *P*. *noctiluca* and *P*. *periphylla*, whose planulae directly develop into ephyrae or medusae, are larger than those of metagenetic scyphozoans (Table 1). This finding that egg sizes are closely related to seasonal alternation of the ontogenetic development of *A*. *coerulea* is in accordance with the hypothesis of Berrill. The number of planulae per medusa did not show significant difference between the direct-development and metagenetic periods, suggesting that the relationship between the size and number of eggs cannot be explained by the well-known trade-off [67–69]. However, the rate of egg production is more important than the number of existing eggs in population dynamics. Information on the turnover rate of eggs is necessary for a strict comparison of egg production rate between the direct-development and metagenetic periods.

**Table 1:** Life cycle type of Scyphozoa in relation to their egg sizes.

Order	Species	Life cycle type	Egg diameter (μm)	References
**Coronatae**	*Nausithoe punctata*	Metagenetic	230	Komai [70]
	*Nausithoe aurea*	Metagenetic	132–208	Morandini & Silveira [71]
	*Nausithoe marginata*	Metagenetic	230	Metschnikoff [72]
	*Linuche unguiculate*	Metagenetic	240	Conklin [73]
	*Periphylla periphylla*	Direct development	1280–1680	Jarms et al. [74]
**Rhizostomae**	*Mastigias papua*	Metagenetic	100	Uchida [75]
	*Cotylorhiza tuberculate*	Metagenetic	120	Goette [76]
	*Nemopilema nomurai*	Metagenetic	100–112	Ikeda et al. [77]
**Semaeostomae**	*Aurelia* sp.	Metagenetic	150–230	Hyde [78]
	*Aurelia coerulea*	Metagenetic	213*	Present study
		Direct development	424*	Present study
	*Pelagia noctiluca*	Direct development	300	Goette [79]
	*Chrysaora hysoscella*	Metagenetic	47–130	Hadzi [80] and Teissier [81]
	*Chrysaora quinquecirrha*	Metagenetic	70–190	Littleford [82]
	*Cyanea capillata*	Metagenetic	60–150	Hyde [78] and Hargitt & Hargitt [83]

*: Mean diameter of the top 5% of oocytes.

### Adaptive significances of the two life cycle types

Different life cycles generally have different adaptive significances. To discuss the adaptive significances of the two life cycle types of *A*. *coerulea* in Maizuru Bay, we focus on the temporal overlapping of ephyra liberation, the seasonal separation of spawning and difference in the duration of the sessile stages (sessile duration) between the two life cycle types.

Both life cycle types are suggested to produce ephyrae from winter to spring (Fig 11). In addition, the ephyrae produced from the direct-development type life cycle (2.5 mm in TBB) were similar in size to ephyrae produced from the strobilation of the metagenetic *A*. *coerulea* (2.1–3.7 mm, [54,84]). Thus, the performances of newly liberated ephyrae in survival are probably comparable between the two life cycle types. In winter to spring, the copepod biomass was high in Maizuru Bay (Fig 3), similarly to other regions [85]. The ephyrae of *A*. *coerulea* show higher growth under higher prey availability [86], and the rapid growth during the ephyra stage is suggested to decrease mortality from predation [84,87]. Therefore, *A*. *coerulea* may produce ephyrae under the abundant prey condition to decrease mortality in the ephyra stage with the rapid growth.

In the metagenetic life cycle, *A*. *coerulea* spawns in the warmer season, and then the polyps attach themselves to substrates for several months to a half year until winter strobilation (Fig 11). Warmer conditions are favorable for polyps to increase their number through asexual reproduction, while, under colder conditions, polyps tend to allocate ingested energy for somatic growth to prepare for the following strobilation [15]. Thus, the summer spawning of metagenetic *A*. *coerulea* would be adaptive in terms of increasing their population size. On the other hand, the metagenetic life cycle would face higher risks of mortality than the direct-development life cycle. During the polyp stage, the major causes of mortality are predation and spatial competition. Numerous marine animals are known to prey on *Aurelia* spp. polyps [88–92]. The polyps also face spatial competition against other sessile animals, such as the mussel *Mytilus galloprovincialis*, which can eliminate polyps of *A*. *coerulea* [57]. The risks of predation and spatial competition during the polyp stage increase as the sessile duration increases, while the sessile duration of *A*. *coerulea* in the metagenetic life cycle (several months to a half year) is much longer than that of *A*. *coerulea* in the direct-development life cycle (less than half a month; Fig 6). Therefore, the summer spawning of metagenetic *A*. *coerulea* is suggested to have an advantage in increasing their population size through asexual reproduction and have risks of predation and spatial competition (Fig 11).

In comparison to the metagenetic life cycle, the direct-development life cycle has a disadvantage in increasing their population size in the short term because of a lack of asexual reproduction during the polyp stage. The winter spawning of the direct-development life cycle may benefit from a lower mortality. As planulae of *A*. *coerulea* are lecithotrophic, they die naturally unless they settle down to suitable substrates before they run out of their energy storage. In other words, a longer longevity of planula leads a higher potential to encounter suitable substrates, although there are some risks of predation and dispersion during the planktonic period. The sizes of planulae of three pocilloporid coral species show intraspecific positive correlation with longevity [93]. In addition, the planktonic larval duration of marine animals generally increases as the temperature decreases [94]. Thus, planulae in the direct-development life cycle, which are large and produced in winter, can have a longer longevity and thus a higher survival rate than those in the metagenetic life cycle. In addition, the shorter sessile duration of *A*. *coerulea* in the direct-development life cycle until ephyra liberation plays an important role in producing ephyrae in an appropriate season. Under the cold condition of 15°C, *A*. *coerulea* polyps do not transform into strobilae within five months from the settlement of planulae [95]. Thus, in the case of the winter spawning, a direct-development life cycle with a shorter sessile duration would be necessary for *A*. *coerulea* to produce ephyrae in winter to spring with high prey availability. Therefore, the direct-development life cycle plays an important role in producing ephyrae in the appropriate season, and potentially decreases the mortality of planulae both from predation and natural death. However, the direct-development life cycle also has a disadvantage in increasing the population size in the short term (Fig 11).

## Conclusions

While asexual reproduction in the metagenetic life cycle of *A*. *coerulea* has been recognized as a key reason for their outbreaks, our results prompt a rethink of its reproductive strategies. Our study clarified that in Maizuru Bay, *A*. *coerulea* is capable to alternate two strategies of reproduction in response to environmental conditions. These alternative life cycle strategies may play important roles in adapting to various environmental conditions, as have been suggested for other organisms (reviewed in [49]). From the present study, the overwintering of mature medusae is suggested to be an indicator of the direct-development life cycle. The direct development of *Aurelia* spp. has been reported from only a few regions [12,19–21]; this may be because most studies concerning the medusa stage have been conducted in warmer seasons. To clarify the importance of the direct-development life cycle in the population dynamics of *Aurelia* spp., further investigations of the mechanisms of overwintering and the generality of the direct development among overwintering populations are required. As Helm et al. [24] suggested, the alternation of two types of life cycles of *A*. *coerulea* in Maizuru Bay could be a suitable model for studying the evolution of direct-development life cycles in scyphozoan jellyfish.

## Supporting information

S1 File Raw data for each figure. The raw data are shown on each sheet.
(XLSX)
