# Correction: Seasonal alternation of the ontogenetic development of the moon jellyfish *Aurelia coerulea* in Maizuru Bay, Japan

**DOI:** 10.1371/journal.pone.0228841

**Published:** 2020-02-03

**Authors:** The PLOS ONE Staff

Scale bars are missing in Fig 4A–4F. The publisher apologizes for the error. Please view the correct Fig 4 here.

**Fig 4:**
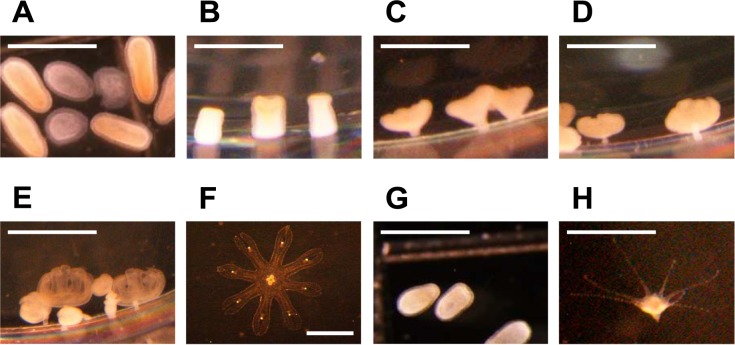
Two types of ontogenetic development of planulae in Maizuru Bay. Transformation process of the direct-development type from planula (A) to ephyra (F). The images B–F were taken 2, 6, 10, 12, and 15 days after the onset of incubation at 10°C, respectively. Transformation process of the metagenetic type from planula (G) to polyp (H). The image H was taken 7 days after the onset of incubation at 28°C. In B–E and H, *A*. *coerulea* attached to the wall of 24-well plates located in the lower part of the pictures. The scale bars indicate 1 mm.
